# The personalized Berger method is usable to solve the problem of tibial rotation

**DOI:** 10.1186/s40634-021-00432-0

**Published:** 2021-12-11

**Authors:** Gömöri András, Gábor Németh, Csaba Zsolt Oláh, Gábor Lénárt, Zsanett Drén, Miklós Papp

**Affiliations:** 1 Department of Traumatology, Semmelweis University – Medicine and Health Sciences, Borsod-Abaúj-Zemplén County Hospital, Üllői út 26., Budapest, 1085 Hungary; 2 Department of Ophthalmology, Borsod-Abaúj-Zemplén County Hospital, Szentpéteri kapu 72-76, Miskolc, 3526 Hungary; 3 Department of Neurosurgery, Borsod-Abaúj-Zemplén County Hospital, Szentpéteri kapu 72-76, Miskolc, 3526 Hungary; 4 Department of Radiology, Borsod-Abaúj-Zemplén County Hospital, Szentpéteri kapu 72-76, Miskolc, 3526 Hungary; 5 TritonLife Róbert Magánkórház, Department of Orthopaedics, Miskolci Egyetem, Egészségtudományi kar, Egyetemváros, Miskolc, 3515 Hungary

**Keywords:** Tibial component malrotation, Revision TKR, Revision planning, Revised Berger method, Personalized Berger method

## Abstract

**Purpose:**

The revision of any total knee replacement is carried out in a significant number of cases, due to the excessive internal rotation of the tibial component. The goal was to develop a personalized method, using only the geometric parameters of the tibia, without the femoral guidelines, to calculate the postoperative rotational position of tibial component malrotation within a tolerable error threshold in every case.

**Methods:**

Preoperative CT scans of eighty-five osteoarthritic knees were examined by three independent medical doctors twice over 7 weeks.

The geometric centre of the tibia was produced by the ellipse annotation drawn 8 mm below the tibial plateau, the sagittal and frontal axes of the ellipse were transposed to the slice of the tibial tuberosity. With the usage of several guide lines, a right triangle was drawn within which the personalized Berger angle was calculated.

**Results:**

A very good intra-observer (0.89-0.925) and inter-observer (0.874) intra-class correlation coefficient (ICC) was achieved. Even if the average of the personalized Berger values were similar to the original 18° (18.32° in our case), only 70.6% of the patients are between the clinically tolerable thresholds (12.2° and 23.8°).

**Conclusion:**

The method, measured on the preoperative CT scans, is capable of calculating the required correction during the planning of revision arthroplasties which are necessary due to the tibial component malrotation. The personalized Berger angle isn’t altered during arthroplasty, this way it determines which one of the anterior reference points of the tibia (medial 1/3 or the tip of the tibial tuberosity, medial border or 1/6 or 1/3 or the centre of the patellar tendon) can be used during the positioning of the tibial component.

**Level of evidence:**

Level II, Diagnostic Study (Methodological Study)**.**

## Introduction

Total knee replacement (TKR) is one of the most successful orthopaedic procedures, with a related satisfaction rate of up to 90%. However, certain patients still complain of persistent pain, contractures or instability. As a result of these complaints, revision arthroplasty could be necessary. The most common causes of revision arthroplasties are as follows: aseptic loosening, pain, complaints regarding patellofemoral pathology, infection, instability, stiff knee, malposition of the components, and as a subgroup, the malrotation of these, primarily the internal rotation of the tibial component [2, 9, 15, 17, 18, 24, 35–38, 40, 45].

The excessive internal and external rotation of the prosthesis components can only be tolerated within a limited range (two to eight degrees). The rotation of the components was determined by several means: the Berger method; using the epicondylar axis, the tibial tuberosity and The Akagi line. Should a revision be required (after ruling out other causes), due to the tibial component malrotation, it is vitally important to determine the extent of the required correction, using high reliability and low variability method [9, 11, 13, 16, 27, 28, 33–35, 38, 44, 46, 49].

The main goal of the research was to elaborate a method with which it is possible to measure the exact rotational position of the tibial component in the case of every patient including the first operation and the possible revision arthroplasty as well.

Hypothesis: The personalized Berger method is capable to choose the anterior reference point as the landmark for the rotational position of the tibial component for most of the patients within a tolerable error threshold.

## Methods

This is a methodological study. The design is mainly descriptive. Systematic sampling was used whenever possible. Data were collected retrospectively.

In a pilot study, the method was designed for CT scans made after TKR. The method uses only reference points of the tibia, this way the measurements before and after TKR are comparable without any change. To achieve a higher number of cases for statistical analysis, we used CT scans from the preparation of patients for TKR.

Eighty-five osteoarthritic knees (of ninety-one consecutive patients) were investigated before undergoing primary TKR at our institution. (01.01.2019.-31.12.2019.) The group consisted of 17 men and 68 women. The mean age was 68.7, with a range of 43-84 years and a standard deviation (SD) of 7.6. Individuals with posttraumatic osteoarthritis of the knee (six people) were excluded from the study.

The measurements were evaluated by the examiners following this guidance:

a transverse plane 2D CT (Model: SOMATOM Definition AS, SYNGOCT2012B, Siemens; 40 Liberty Boulevard Malvern, PA 19355, United States of America) was administered on the affected knee joints, and the measurements were taken in two slices: firstly 8 mm below the proximal tibial joint surface, where the proximal resection would take place; secondly on the slice which contains the TT and the patellar tendon reaches the periosteum.

For the following process, a DICOM viewer was used. (Model: JiveX 5.0.6.10 RC01, VISUS health IT GmbH).

**Fig. 1:**
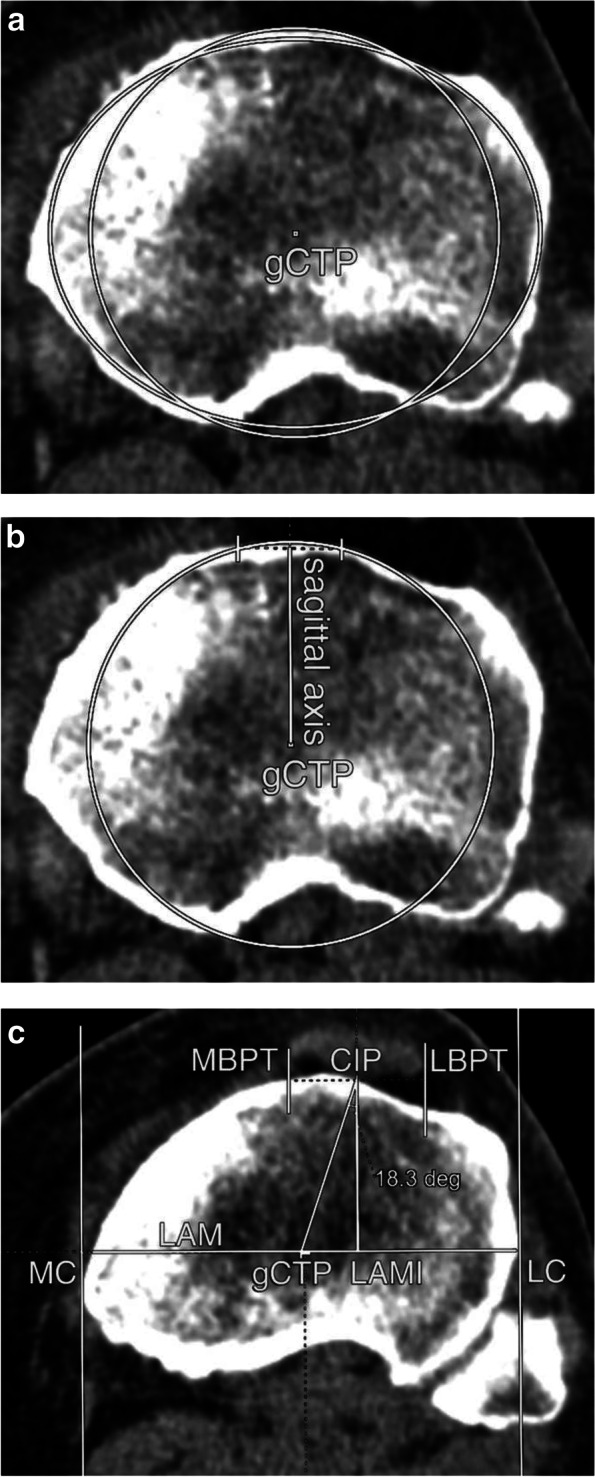
**A** The geometric centre of the tibial plateau (gCTP), obtained per the ellipse tool, was used as the centre of a circle. **B** The circle and the anterior cortex had two intersections. The section between these points was halved and the latter was connected to the gCTP, providing the sagittal axis of the tibial metaphysis. **C** The line perpendicular to the former and running through the gCTP, was the longer axis of the metaphysis (LAM). The gCTP, the sagittal axis and the LAM were transposed to the slice containing the periosteum-ligament connection. Two lines were created parallel to the sagittal axis and tangential to the medial (MBPT) and the lateral (LBPT) border of the patellar tendon. A perpendicular line was drawn to the MBPT and the LBPT, tangential to the midpoint of the MBPT and the LBPT, whose intersection with the anterior cortex was determined (CIP). A perpendicular line was emitted from the CIP to the LAM, and the intersection with the LAM was named LAMI. The angle measurement tool was used to evaluate the gCTP-CIP-LAMI angle

The gCTP was determined by the ellipse annotation to obtain the most achievable cortical coverage on the slice, which is 8 mm below the tibial plateau. (Fig. 1A) The gCTP, obtained per the ellipse tool, was used as the centre of a series of circles, laid on the tibial metaphysis to create the sagittal axis of the tibia. That which just exceeds the anterior cortex was chosen from the concentric circles; in this way, the circle and the anterior cortex had two intersections. The section between these points was halved and the latter was connected to the gCTP, providing the sagittal axis of the tibial metaphysis. (Fig. 1B) The line perpendicular to the former and running through the gCTP was the longer axis of the metaphysis (LAM). The intersections of the long axis as well as the medial cortex (MC) and the lateral cortex (LC) were located. The LC-MC distance is the greatest crosswise width of the tibia. The gCTP, the sagittal axis and the MC-LC axis were transposed to the slice containing the periosteum-ligament connection. Two lines were created parallel to the sagittal axis and tangential to the medial (MBPT) and the lateral (LBPT) border of the patellar tendon. A perpendicular line was drawn to the MBPT and the LBPT, tangential to the midpoint of the MBPT and the LBPT, whose intersection with the anterior cortex was determined (CIP). The original Berger method connected the gCTP and the TTT; however, we connected the gCTP and the CIP, as we found it difficult to identify the TTT in certain cases. A perpendicular line was emitted from the CIP to the LAM, and the intersection with the LAM was named LAMI. A right triangle was constructed (gCTP-LAMI-CIP); the hypotenuse was the gCTP-CIP segment, the short leg was the gCTP-LAMI segment and the long leg was the LAMI-CIP segment. The angle measurement tool was used to evaluate the angle between the LAMI-CIP and the gCTP-CIP. (Fig. 1C).

The measurements were conducted by an independent neurosurgeon (Cs. O.) and two radiologists (Zs. D. and G. L.) twice per examiner. The measurements were taken 6 weeks apart. All patient data were made anonymous. Furthermore, the examiners were provided with a code before carrying out the statistical analysis, to counteract any bias based on personal acquaintance.

The calculation was made to show what portion of the examined knees fits the following ranges: ±3°according to Tandogan et al. (12.2°-23.8°); ±5.8° according to Bell et al. [11, 49].

The statistical analysis was performed using MedCalc version 13.3.3.0 (MedCalc Inc., Ostend, Belgium). The data were described as mean, SD and ranges. The coefficient of variation was defined as the ratio of the standard deviation to the mean (as a percentage). Repeatability was described in three ways. The first method was the intra-session test-retest variability (also known as repeatability or limits of repeatability). This intra-session test-retest variability was calculated by multiplying the SD by 2.77 and indicated the estimate of the limits within which 95% of measurements for the same subject should be recorded. The second method comprised the intra-observer coefficients of variation (CV), expressed as a percentage and the third method involved the intra-class correlation coefficient (ICC), which verifies the reliability of the measurements. In addition, Cronbach’s alpha gauged the internal consistency of the measurements. The inter-rater reproducibility was calculated using the inter-observer CV and the inter-observer ICC. The ICC values ranged from 0 to 1 and were classified as follows: an ICC value of 1 indicates perfect reliability, 0.8 to 1 is very good, 0.61 to 0.8 is good, 0.41 to 0.6 is moderate and 0.4 or less is poor [24].

An institutional review board approved the study (protocol number BORS-05/2021, date of approval 05.02.2021).

## Results

The following descriptive data can be provided, based on the work of the three examiners: the mean of the personalized Berger angle was 19.5° for Cs. O., with an SD of 6.3°, giving a CV of 32.3%. The range of the measurements was 2.1°-31.4°. The same data for G. L. were: 17.9° mean with 6.4° SD and 35.5% CV. The range was between 1.2° and 37.9°. The measurements of Zs. D. indicated a mean of 17.3°, with 6.0° SD, giving a 35.0% CV, with a range of 2.6°-30.3°.

Repeatability was calculated by three distinct methods. The ICC values were 0.93 for Cs. O, 0.89 for G.L. and 0.90 for Zs. D., indicating very good results. The intra-session test-retest variability was 3.3° for Cs. O., 4.2° for G.L. and 4.3° for Zs. D. The CV was 7.5%, 10.9% and 10.85%, respectively. Cronbach’s alpha showed 0.96, 0.94 and 0.95, respectively.

Inter-rater reproducibility was calculated by two methods. Inter-observer CV was 11.6%, while inter-observer ICC was 0.87, which constitutes a very good result.

Taking all the measurements into account, regarding the personalized Berger angle we can conclude that only 45.9% of the cases were within the average ± 3° and 70.6% were within the average range of ±5.8°.

## Discussion

Through the personalized Berger method, the rotational position of the tibial component is achievable for every patient including both TKR and revision arthroplasty.

The optimal rotational position of the tibial component was found to be crucial, as its excessive internal rotation can cause: patellar maltracking, patellofemoral instability, persistent anterior pain, flexion and mid flexion tibiofemoral instability, early wear of the polyethylene (PE) component, early loosening, stiffness and contracture. Excessive internal rotation of the tibial component can cause an extension deficit [1, 3, 9–13, 16–19, 21, 25, 33, 41, 44, 47, 49].

There is conflicting data relating to the clinically tolerable limits of tibial component internal malrotation. According to Nicoll, the internal rotation error of the tibial component by 8° is tolerable, while Barrack reports 6°; Bell showed that more than 5.8° of internal rotation cause anterior pain of the knee. All of the studies used the classical Berger method [9, 19, 38, 44, 49].

According to Kim, less than 2° of external rotation constitutes a risk of component failure. Malrotation of the components plays a significant role in early TKR failure. Should this cause pain, early revision surgery may become inevitable. Before revision surgery is selected, the cause should be identified. If revision surgery is likely, due to component malrotation - taking into account the limited range of the tolerable angle - an accurate and repeatable measuring method is needed to specify the degree of correction [13, 16, 27, 28, 33–35, 46].

Rotation and malrotation of the femoral component are measurable on a single axial CT, referenced to the sTEA. Measurement of the rotation of the tibial component is more complicated, therefore, several methods are recommended, using various anatomical reference points, with the help of 2D and 3D CT.

Usually, the TT, the MBPT or its medial 1/6 or 1/3, the centre of the TT or that of the patellar tendon, are used as the anterior reference point. The anterior tibial curved cortex is used by Baldini, Indelli and Kim. The centre of the PCL, the centre of the greatest mediolateral width of the tibia, the centre of the posterior tibial margin axis, the centre of the sTEA transposed to the tibia or the gCTP, are used as the posterior reference point [4, 5, 7, 8, 12, 14, 20, 22, 23, 26–35, 44, 48, 49].

The accuracy and the repeatability of 15 different tibial axes were evaluated by Saffarini et al. in a systematic review. The angle between the perpendicular line to the sTEA and the original Akagi line was the most consistent. The methods using the gCTP as the posterior reference point showed the best inter-observer repeatability. However, the minimum and the maximum values of the angulations, compared to the perpendicular line to the sTEA, significantly exceeded the thresholds of clinical relevance. A difference of less than ±5° between the line connecting the gCTP and the medial 1/3 of the TT and the line perpendicular to the sTEA was found in 67.5% of cases; a difference between the line connecting the gCTP and the medial border of the TT and the line perpendicular to the sTEA was found by Lützner in only 3.8% [4, 5, 8, 14, 20, 22, 27, 29–31, 35, 39, 43, 44].

Rotation between the femoral and tibial components is restricted by the fixed bearing insert unpredictably, therefore, the angle between the sTEA or the line perpendicular to it, and the axis of the tibial component, should be evaluated critically. In this way, a method should be chosen which uses solely the landmarks of the tibia. The centre of the PCL and the Akagi line, which is seen as a gold standard, are difficult to identify on the 2D CT, following TKR [4, 5, 35].

As a result of the above, the gCTP can be used as the posterior, while certain specified points of the TT or parts of the patellar tendon can be used as the anterior reference points, meeting the recommendations of other authors. The Berger method, which evaluates the rotation of the femoral and tibial components, depends upon the gCTP-TTT distance; therefore its value varies from patient to patient [12].

High variability of the mediolateral localization of the TT was realized by Howell (32-47 mm between the medial border of the TT and the MC of the tibia); 70% of cases were outside the range of the clinically relevant threshold while using the gCTP as the posterior reference point. This means that in the vast majority of cases, the difference was more than the clinically significant threshold. In the case of 45.9% of the patients, the value was ±3° and 70.6% of the population, measured using our method, were within the ±5.8° range [27, 35].

**Fig. 2:**
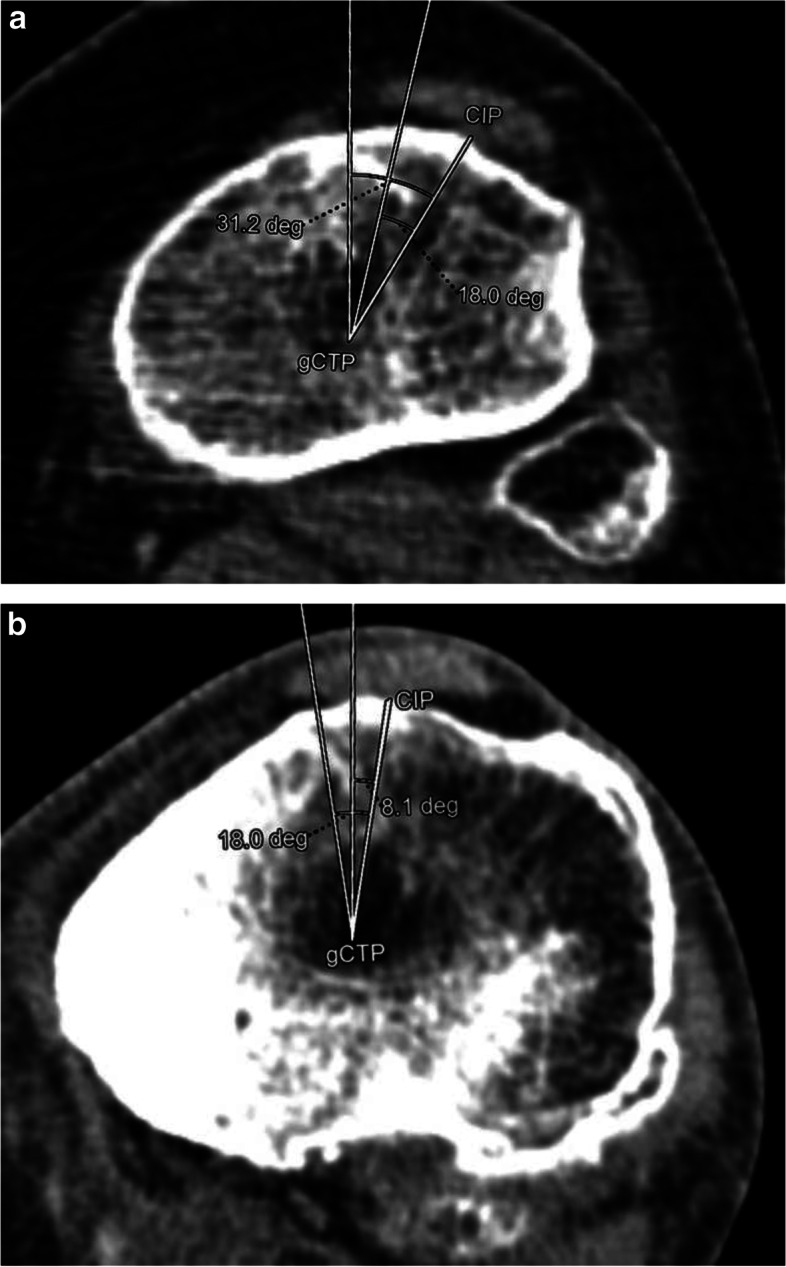
The role of the position of TTT (or CIP) can be seen. Comparison of the classical and the personalised Berger angle are shown on both of the images; **A** Lateralised TTT; **B** Medial TTT

Simply measuring 18 degrees to the line between gCTP and TTT (or CIP) can lead to severe rotational malposition. (Fig. 2A, B).

If the method could be carried out with acceptable reliability and repeatability on the preoperative CT scans, then the degree of the correction, carried out on account of the tibial component malrotation can be defined precisely [6, 8, 12, 20, 23, 25, 26, 29–31, 34, 35, 44].

The repeatability and reliability of the classic Berger method and its modifications were examined by several authors. Most of them mention good and excellent intra- and inter-observer intra-class correlation coefficients (ICC) [8, 23, 26, 27, 29–31, 35, 42, 44, 48].

Even if most of the authors found good and very good intra and inter-observer correlation, the minimum and the maximum values of the angulations, compared to the perpendicular line to the sTEA, significantly exceeded the thresholds of clinical relevance [8, 14, 20, 22, 35, 44].

The Berger method was improved by us to achieve better repeatability. Following an analysis of our experiences, we concluded that the centre of the TT is difficult to identify accurately on a 2D axial CT, which concurs with the findings of Saffi. The method proposed by us can be implemented in any institute using CT scans; any software that automatically enables the ellipse can optimize the ICC even further [12, 44].

Our results are amongst the best, with similar inter- and intra-observer reliability compared to other authors; for this, we have the following explanations: the reference points were specified in a precise manner and all three of the examiners followed the same protocol [6, 8, 22, 23, 26, 27, 29–31, 34, 35, 39, 42, 44, 48].

Concerning our study and the method proposed by us, there are certain limitations. The case number was relatively low with only three examiners. Even so, the multiplicity for statistical analysis was satisfactory. CT scans from knees awaiting TKR were evaluated to obtain the data for our method, required to plan revision arthroplasties. The personalized Berger method was developed on CT scans with metal subtraction of patients awaiting revision arthroplasty. The number of the cases was not satisfactory for the research of reliability in the case of postoperative CT scans; therefore preoperative CT scans were used in further stages of the study.

## Conclusion

Using the aforementioned method with simple geometric principles, the personalized Berger value is achievable without the use of any femoral references. The inter- and intra-observer ICC values in the “very good” range render this method viable for use in evaluating the required correction in the case of planned revisions, due to tibial component malrotation, while respecting the relevant clinical thresholds. The personalized Berger angle isn’t altered during arthroplasty, this way it determines which one of the anterior reference points of the tibia (medial 1/3 or the tip of the tibial tuberosity, medial border or 1/6 or 1/3 or the centre of the patellar tendon) can be used during the positioning of the tibial component.

## Abbreviations

CIP: Intersection with the anterior cortex (Cortex Intersection, Patellar)
CT: Computed Tomography
CV: Coefficients of Variation
DICOM: Digital Imaging and Communications in Medicine
gCTP: Geometric Centre of the Tibial Plateau
ICC: Intra-class Correlation Coefficient
LAM: Longer Axis of the Metaphysis
LAMI: Longer Axis of the Metaphysis Intersection
LBPT: Lateral Border of the Patellar Tendon
LC: Lateral Cortex
MBPT: Medial Border of the Patellar Tendon
MC: Medial Cortex
PCL: Posterior cruciate ligament
SD: Standard Deviation
sTEA: Surgical Transepicondylar Axis
TKR: Total Knee Replacement
TT: Tibial Tuberosity
TTT: Tip of the Tibial Tuberosity
